# The use of X-ray computed tomography for advanced detection of *Globodera pallida*

**DOI:** 10.1371/journal.ppat.1012753

**Published:** 2025-08-22

**Authors:** Eric C. Pereira, Christopher A. Bell, Peter E. Urwin, Saoirse Tracy

**Affiliations:** 1 School of Agriculture and Food Science, University College Dublin, Belfield, Dublin, Ireland; 2 School of Biology, Faculty of Biological Sciences, University of Leeds, Leeds, United Kingdom; University of California, Davis Genome Center, UNITED STATES OF AMERICA

## Abstract

Potato cyst nematodes (PCN), namely *Globodera pallida*, pose a significant threat to potato production worldwide. Accurately quantifying nematode population densities is crucial for effective management strategies. However, traditional detection methods are time-consuming and often imprecise. X-ray computed tomography (CT) offers high-resolution, non-destructive and three-dimensional (3D) imaging frequently used to visualise internal structures of objects, materials or biological tissues, allowing for detailed analysis of their composition, defects, and morphology. Here, we demonstrate the effectiveness of X-ray CT in detecting and quantifying PCN cysts in two different soil types. The ability to achieve 3D imaging and volume quantification of PCN cysts from soils allows accurate enumeration of their egg content. Combined with the potential to co-analyse other organisms within the same sample, we propose X-ray CT as an innovative tool for comprehensive soil health assessments and sustainable pest management in agriculture.

## Author summary

Potato cyst nematodes pose a major threat to global crop production, causing significant losses. Traditional detection methods are often challenging, time-consuming and imprecise. Our study explores a new approach using X-ray computed tomography (CT), a powerful imaging tool that creates detailed 3D images *in situ*. X-ray CT can be used to detect and measure cysts nematodes, even within different soil types. This detailed analysis allows us to determine the cyst structures and estimate the eggs contained. This method could also be expanded to study other soil organisms, making it a valuable tool for monitoring soil health and improving agricultural pest management.

## Introduction

X-ray computed tomography (CT) has emerged as a promising tool in soil science due to its non-invasive, high-resolution 3D imaging [1], offering unprecedented insights into soil pores netwoks [2,3] that soil-borne organisms may occupy. Variations in X-ray attenuation, which correlate with material density and composition, differentiate structures, allowing visualisation of soil architecture and biological components.

In this study, we demonstrate the potential for deployment of X-ray CT to investigate soil pathogens, focusing on plant-parasitic nematodes as a case study. Every major crop species is host at least one plant-parasitic nematode species, resulting in over $470M in global crop losses daily [4]. *Globodera pallida* is an obligate sedentary parasite of Solanaceous plants, particularly potato. The lack of overt, specific symptoms and the reliance on labour-intensive sampling leads to an underestimation of its impact. Efficient sampling requires multiple soil collection, introducing variability and often facing seasonal and environmental constraints [5]. Traditional methods, such as flotation, elutriation, and microscopy, are labour-intensive, time-consuming and limited in scope [6]. *G. pallida* cysts exhibit a distinct size, shape, and internal density, making X-ray CT particularly well-suited for their non-destructive identification and analysis within soil samples.

Automating cyst quantification from soil will help to increase efficiency within the system, providing more comprehensive coverage of agricultural soils. An alternative technique that is rapid, efficient, and accurate would improve advisory procedures. Reliable quantification of *G. pallida* populations directly influences control strategies. Increasing nematicide restrictions [7] have intensified the need for precise diagnostics, aligned with legislative efforts like the European Council Directive and North American Plant Protection Organisation regulations. The European and Mediterranean Plant Protection Organisation [8] advises *G. pallida* cyst extraction protocols, emphasising the importance of accurate quantification and viability assessment for effective management [9]. Nevertheless, *G. pallida* infestations continue to rise globally [10]. Early detection and identification of pathogens are crucial to mitigate agricultural losses, necessitating a paradigm shift in detection methodologies. Precise quantification is crucial for determining infestation levels, guiding treatment decisions, and monitoring control strategies. Once optimised to enable high-throughput analysis, X-ray CT presents an exciting alternative to traditional methods.

Here, we apply this approach to identifying and quantifying *G. pallida* in two different soil types, providing evidence for the capability of X-ray CT in soil pathogen detection. Importantly, this method scans the entire sample rather than isolating cysts for analysis. With further optimisation, this approach could enable rapid multi-pathogen diagnostics and improve understanding of soil factors that affect pathogen communities.

## Results

### Cyst characterisation by X-ray CT

**Fig 1:**
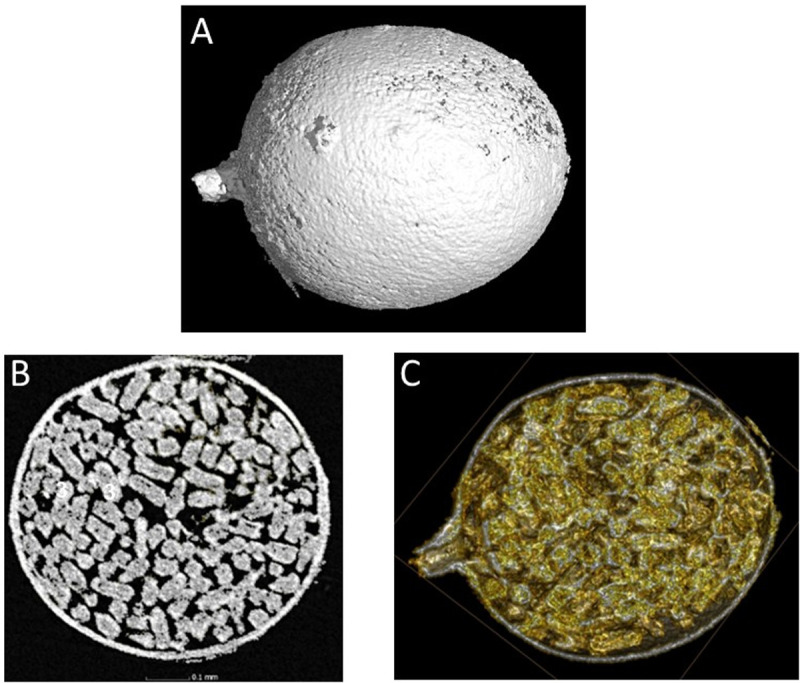
Xray Computed Tomography scanning of a *Globodera pallida* cyst. *G. pallida* cysts were scanned via X-ray CT to visualise the cuticle (A) and interior contents from 360^0^ (B, C). Cross-sectional photos at 50% depth show distinct egg profiles (B, C).

X-ray analysis enabled detailed visualisation of *G. pallida* cysts externally and internally (Fig 1). Hydration influenced density contrast and feature visibility. Dry cysts possessed a dehydrated, less permeable cuticle, exhibited by a stronger density contrast between the cyst wall and its surroundings but reduced visibility of internal contents (Fig 1A). Wet cysts retained internal hydration, allowing clear visualisation of eggs while reducing density contrast between wall and its contents, thereby facilitating clearer visualisation of individual eggs within the cavity (Fig 1B and 1C). Cysts displayed a structurally sound outer cuticle and an egg-filled inner cavity with residual empty spaces, indicating a strong and intact structure (S1 Animation). Note that hydration does not indicate viability, and that cysts from both soils were viable.

### Identification of *G. pallida* in soil by X-ray CT

**Fig 2:**
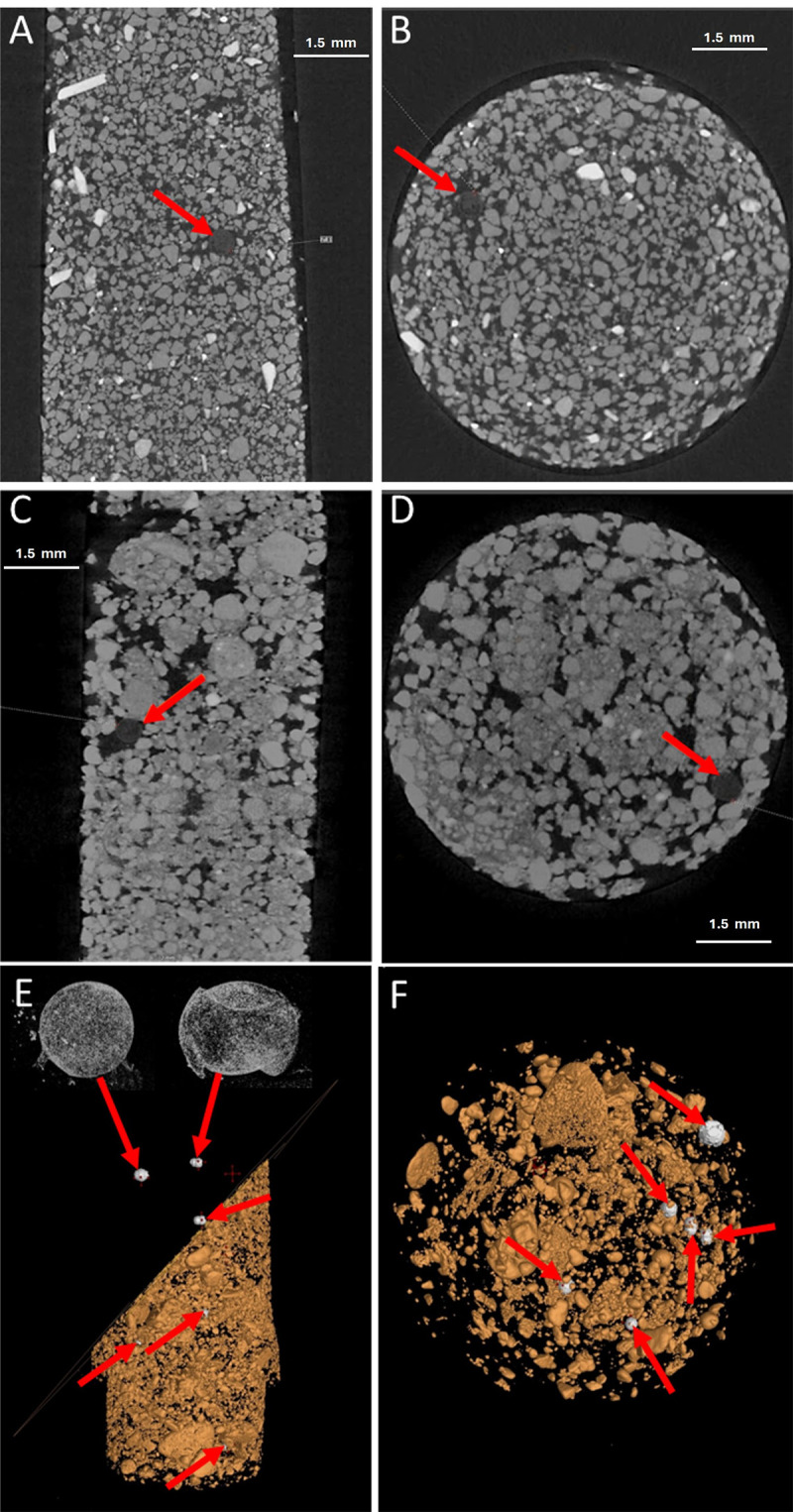
Xray Computed Tomography scanning of soil containing *Globodera pallida* cysts. Sand (A, B) and loamy soil (C, D, E, F) were spiked with *G. pallida* cysts (red arrows) and scanned via X-ray CT. Soils were scanned in 15 ml Falcon tubes and visualised as either side (A, C, E) or top-down (B, D, F) profiles. Computer-led subtraction of soil (E) allowed distinct separation and quantification of cysts from the soil.

Soil samples were artificially spiked by manually introducing a known number of cysts into soil matrices. This approach simulated natural infestation conditions, allowing for the controlled evaluation of detection accuracy. *G. pallida* cysts were confirmed in both sandy (Fig 2A and 2B) and loamy (Fig 2C and 2D) soils, despite inherent contrasts in soil composition, characterised by distinct textures and varying porosity levels. Cysts were identified based on density contrasts and morphological characteristics, enabling differentiation between damaged and intact cysts through X-ray CT segmentation (Fig 2E and 2F and S2 Animation).

### Correlation between cyst volume and number of eggs

**Fig 3:**
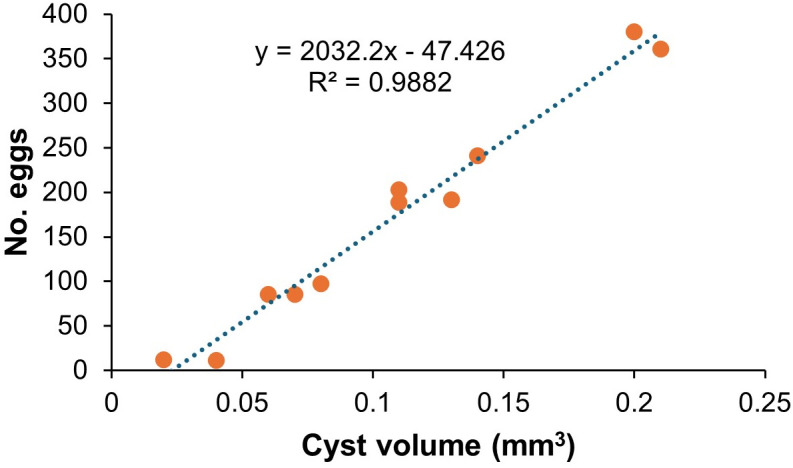
Egg number correlates with X-ray CT determined cyst volume. Cyst volumes were quantified through X-ray CT scanning, and exact egg numbers were determined via microscopy counts. Pearson correlation R^2^ = 0. 9882.

Cyst-projected surface area strongly correlates with egg content [11]. X-ray CT yielded the quantification of cyst volumes by first segmenting the cysts from the surrounding soil matrix based on grayscale intensity differences and calculating the total volume of each segmented cyst by integrating the voxel-based 3D reconstruction, where each voxel represents a discrete unit of volume. The volume of the 11 samples ranged from 0.02 to 0.21 mm³ (S1 Table). A positive correlation between cyst volume and egg count was observed (Pearson correlation analysis coefficient (R) value of 0.9882) (Fig 3). X-ray CT predictions of egg content demonstrated a + /-17% error margin compared to manual counting.

## Discussion

This study provides novel insights into utilising X-ray CT to identify and analyse soil pathogens with real-world utility. *G. pallida* detection was used as a case study due to its significant agricultural relevance. Although X-ray CT has been applied in biological and soil studies [12], its direct use for *in situ* pathogen detection remains underexplored. Previous research focused mainly on nematode morphology and distribution in tree tissues [13,14]. The key advantage of X-ray CT is its significant labour savings over traditional cyst and egg extraction methods, which are time-consuming and require extensive microscopy and expertise [15–17]. In contrast, X-ray CT requires minimal preparation, rapidly generates high-resolution 3D images, and enables (semi)-automated segmentation. While specialised skills are needed for operation and data processing, once optimised, the workflow becomes efficient, with standard protocols for trained users. This technique supports computer-assisted analysis, leveraging X-ray attenuation and machine-learning algorithms for accurate cyst identification, reducing subjectivity and enhancing reproducibility. Moreover, it allows non-destructive, multi-target detection of various soilborne organisms, providing comprehensive insights from intact samples, improving agricultural diagnostics.

The process of using X-ray CT is not only efficient but also highly adaptable, making it a powerful tool for soil pathogen research across various soil types and conditions. A key strength of X-ray CT is its ability to provide detailed 3D imaging of *G. pallida* cysts, offering unprecedented insights into their morphology, structural integrity, and internal organisation. Unlike traditional microscopic techniques, which provide only 2D views and require extensive sample preparation, X-ray CT enables visualisation of intact cysts, revealing internal egg distribution, cuticle thickness, and potential degradation patterns that may indicate cyst viability. These insights are crucial for understanding the physiological states and viability of *G. pallida* [18], aiding in developing effective pathogen management strategies. Moreover, since X-ray CT captures the whole soil structure in detail, the same scans can be used to look for other soilborne organisms. This makes the method useful beyond research, with potential applications in industry for detecting multiple pathogens at once. It could help improve diagnostics and monitoring in agriculture by offering a more efficient, all-in-one solution.

X-ray CT extends the earlier work of Atkinson et al [19], who analysed cross-sectional areas and egg numbers, by enabling precise volumetric measurements of cysts and more accurate egg counts. Our findings reveal a proportional relationship between cyst volume and egg number, indicating that cysts are typically filled rather than containing excess internal space. This suggests an optimised reproductive strategy where cyst size efficiently accommodates developing eggs. Visualising intact cysts also enables assessment of egg distribution, reproductive output variations, and signs of degradation or viability through internal density contrasts. Such non-destructive imaging offers valuable insights into *G. pallida* reproductive potential, cyst maturation and viability, advancing our understanding of its biology and life cycle dynamics.

A transformative aspect of X-ray CT is its potential for automation through artificial intelligence (AI) [20]. AI can be trained to identify cyst images, further streamlining the process and reducing the need for manual intervention. Machine learning algorithms can be developed to enhance the precision of pathogen detection, facilitating rapid and accurate identification of nematode populations.

Beyond *G. pallida*, X-ray CT can detect other soil organisms, making it a valuable tool for comprehensive soil health assessments and sustainable pathogen management. We are currently investigating the utility of the technique in identifying other important soil-borne organisms. While some of these may be too small for direct visualisation, X-ray CT can indirectly detect their effects by visualising changes in root morphology, soil structure and disease symptoms [21,22].

Despite these advantages, X-ray CT still have limitations. Its resolution detects *G. pallida* cysts but may need refinement for smaller pathogens or early-stage nematodes. Future work should focus on improving sensitivity and resolution.

## Conclusion

In conclusion, adopting X-ray CT for soil pathogen detection offers numerous advantages over existing methods. This application extends beyond *G. pallida* to other nematodes and invertebrate pathogens, making it a critical technology for improving our understanding and management of soil-borne threats in agricultural systems. By providing a non-invasive, high-resolution imaging solution, X-ray CT represents a significant advancement in the tools available to researchers and practitioners in nematology and soil science. The use of X-ray CT imaging data to detect *G. pallida* underscores its potential for broader applications in identifying other soil-borne pathogens, reinforcing sustainable agricultural practices, and advancing soil management strategies. In future, by leveraging image processing and pattern recognition, X-ray CT data could automate pathogen identification, quantify soil biodiversity, and assess overall soil health with minimal human intervention.

## Materials and methods

### *Globodera pallida* cyst samples

*G. pallida* (Lindley) cysts were sourced from infected soil provided by the Urwin lab (University of Leeds). The cysts were extracted using the Fenwick can flotation method [23]. 200 g of dry soil was sieved (1 mm) into a water-filled stainless-steel Fenwick can. Continuous water flow facilitated the flotation of cysts, which were collected via side collar onto a 250 µm sieve, transferred to filter paper, and examined under a dissecting microscope (Leica M80, Germany). Cysts were manually counted and stored in sterile distilled water at 4 °C.

For experimental spiking, known cyst numbers were added to RHS Horticultural sandy loam and clay (Baileys of Norfolk, UK) in 15 mL Falcon tubes. Samples were gently mixed for even cyst distribution. After X-ray CT, cysts were crushed in water and examined via light microscopy to assess egg content for correlation with CT data.

### X-ray computed tomography (CT) scanning

*G. pallida* cysts were scanned in both dry and wet conditions to assess the impact of hydration on imaging quality. Dry cysts were air-dried at room temperature for 48 hours prior to scanning. Wet cysts were maintained in water, ensuring retention of internal hydration. A total of 20 cysts, nine to optimise the X-ray CT scanning method and 11 to determine the correlation between volume and egg number, and 20 soil samples were characterised using a GE Nanotom M X-ray CT machine (GE Measurement and Control Solutions, Germany). The v|tome|x M was set at a voltage of 65 kV and a current of 300 μA to optimise contrast between background soil and objects of interest. The ‘Fast Scan option’ achieved a voxel resolution of 1.60 μm. 1,078 projection images were taken per scan at 200 m/s per image. The images were reconstructed using Phoenix datos| × 2 rec reconstruction software, combining the scans into a single 3D volume representing the entire sample.

### Image processing

X-ray CT images were processed in VGStudioMax v3.2 (Volume Graphics GmbH, Heidelberg, Germany). Cysts were segmented using the Region Grower tool by setting seed points and grayscale thresholds to isolate cysts from background soil. Erosion and dilation (1 pixel) were applied to refine boundaries and ensure clear separation from the soil matrix. An image analysis workflow was followed, including adaptive thresholding to distinguish cysts based on density contrast (S1 Fig). Feature extraction was then performed to measure cyst volume, surface area, and internal structure. Volume (mm³) was calculated from voxel counts, and density was estimated using mean grayscale intensity. The distinctive and conserved morphology of cysts was used to assist identification following contrast enhancement. These parameters were used to assess cyst morphology, differentiate intact from collapsed cysts, and estimate egg content based on volume.

Finally, *G. pallida* cysts were classified by size, shape, and internal density patterns as visualised in the X-ray CT images.

## Supporting information

S1 Animation X-ray Computed Tomography (CT) scan of *a Globodera pallida* cyst, revealing its complete internal structure, including the densely packed eggs. (MP4)

S2 Animation X-ray Computed Tomography (CT) scan showing the distribution of *Globodera pallida* cysts within a soil sample. (MP4)

S1 Table Correlation between cyst volume (mm³) and egg count for 11 samples analysed using X-ray CT. (PDF)

S1 Fig Image segmentation workflow for X-ray CT analysis of *Globodera pallida* cysts. (JPG)
