# Supplementary material for: The use of X-ray computed tomography for advanced detection of Globodera pallida
Source: PLoS Pathog. 2025 Aug 22;21(8):e1012753. doi: 10.1371/journal.ppat.1012753 (PMC12404637; doi:10.1371/journal.ppat.1012753)
Supplement: S1 Table — (PDF) [file ppat.1012753.s003.pdf]

Table S1. Correlation of the cyst volume and No. of eggs (n=11).

Sample	Cyst volume (mm$^{3)}$	No. eggs
**S1**	0.02	12
**S2**	0.04	11
**S3**	0.06	85
**S4**	0.07	85
**S5**	0.08	97
**S6**	0.11	189
**S7**	0.11	203
**S8**	0.13	192
**S9**	0.14	241
**S10**	0.2	380
**S11**	0.21	361
