# Supplementary figures and images for: The use of X-ray computed tomography for advanced detection of Globodera pallida
Source: PLoS Pathog. 2025 Aug 22;21(8):e1012753. doi: 10.1371/journal.ppat.1012753 (PMC12404637; doi:10.1371/journal.ppat.1012753)

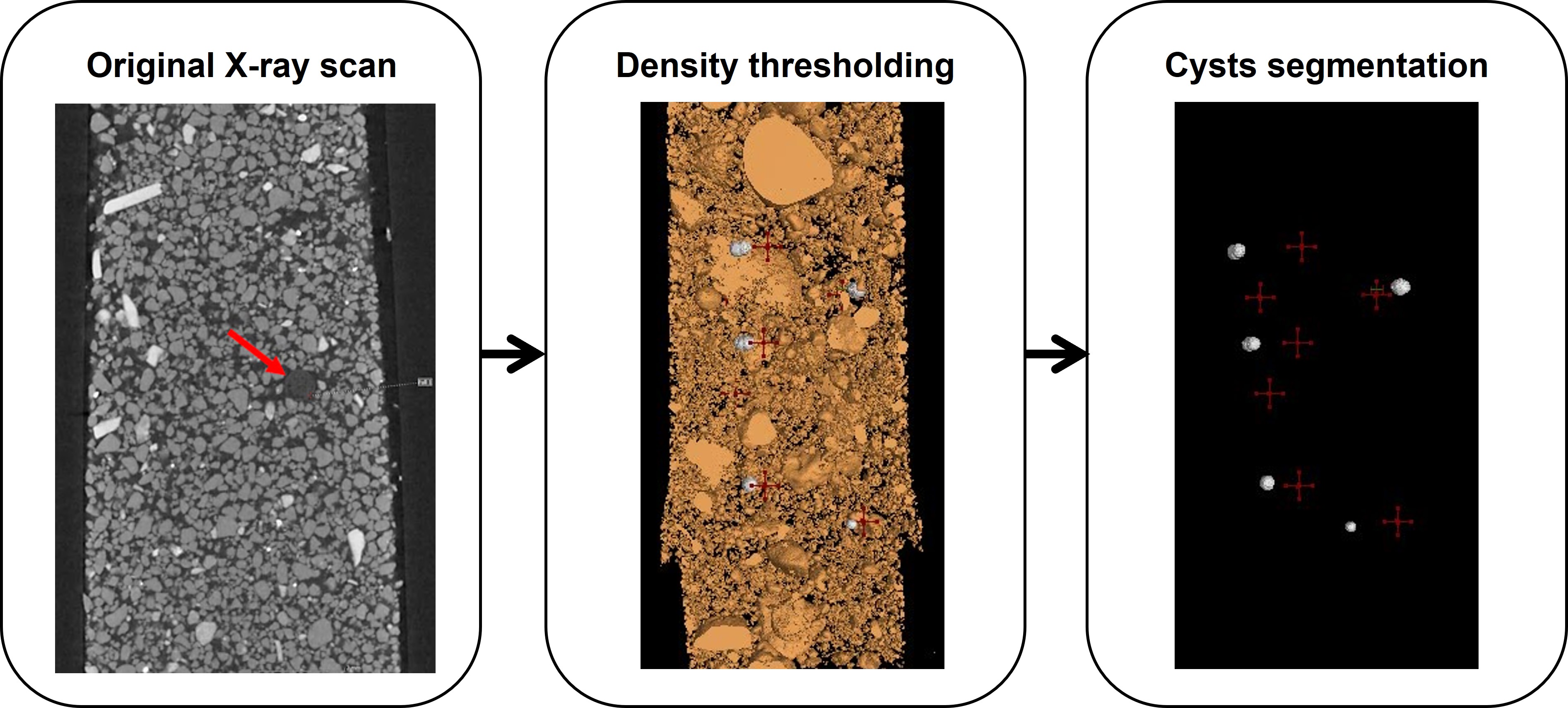

Supplement: S1 Fig — (JPG) [file ppat.1012753.s004.jpg]
